# A Research on Preparation and Performance of Volcanogenic Sand Concrete from the Philippines

**DOI:** 10.3390/ma16062306

**Published:** 2023-03-13

**Authors:** Libo Bian, Jiapeng Bian, Linna Ding, Yangguang Zhao, Haichao Lin, Shaomin Song

**Affiliations:** 1 School of Civil and Traffic Engineering, Beijing University of Civil Engineering and Architecture, Beijing 100044, China; 2 Collaborative Innovation Center for Energy Saving & Emission Reduction and Urban-Rural Sustainable Development in Beijing Co-Established by the People’s Government of Beijing Municipality and the Ministry of Housing and Urban-Rural Development of China, Beijing 100044, China; 3 GC8 KINMING Inc., Zambales 2201, Philippines

**Keywords:** volcanogenic sand, fine aggregate, optimization, concrete, lightweight aggregate

## Abstract

The river sand in the Santo Tomas River area of the Philippines is a kind of volcanogenic sand. The sand is fine sand with a fineness modulus of 2.2, an apparent density of 2380 kg/m^3^, a bulk density of 1320 kg/m^3^, a mud content of 6.7%, a methylene blue value of 1.2, a soluble chloride ion content of 0.00071%, and a light-matter content of up to 12.2%, which does not meet the requirements of the three-zone grading. Based on a series of experiments, this paper systematically studies and compares the workability, mechanical properties, and durability of two kinds of concrete with the river sand in the Santo Tomas River area and natural river sand in Beijing, China as fine aggregates, respectively. In addition, volcanogenic sand in the Philippines is technically optimized for the purpose of in-depth study. After optimization, such sand reaches the standard of Zone II-graded medium sand and is comprehensively improved in performance, which is evidenced by a fineness modulus of 2.4, an apparent density of 2570 kg/m^3^, a bulk density of 1550 kg/m^3^, a light-matter content of 6.0%, and a mud content of 6.7%. Study results show that in terms of mechanical properties, the concrete made of the optimized river sand in the Santo Tomas River area is superior to that made from the natural river sand in the Beijing area. In addition, separated light matter can be used as a natural light aggregate, which has a bulk density of 960 kg/m^3^, a cylindrical compressive strength of 2.5 MPa, and a 1 h water absorption of 8.2%, respectively.

## 1. Introduction

As raw materials, sand and gravel are most frequently used for global infrastructure construction. At present, 40–50 billion tons of sand and gravel are needed globally [1]. Due to their importance, sand and gravel have become the world’s second-largest resource after water. It is estimated that by 2030, the global demand for sand and gravel may increase to 60 billion tons per year [1]. Not long ago, a video conference of the Global Aggregates Information Network (GAIN) was held, at which Michael W. Johnson, President of the National Stone, Sand and Gravel Association (NSSGA), said that the U.S. sand and gravel market was booming in 2021 due to the phase-I implementation of the Infrastructure Investment and Jobs Act (IIJA), and the U.S. building industry would remain very promising by 2026. In addition, Antonis Antoniou Latouros, President of the European Aggregates Association, said that Europe experienced a strong economic recovery in 2021, and the European market of sand and gravel aggregates remains promising overall despite the adverse factors of energy and cost [2]. In 2018, China as the world’s largest producer and consumer of sand and gravel, consumed more than 20 billion tons (accounting for nearly half of the global consumption) of sand and gravel [3]. At the present stage, China is sparing no effort to dredge and improve rivers, stop massive sand mining operations and crack down on illegal sand mining, thereby resulting in a significant reduction in river sand mining [4]. In the next few years, the demand for sand and gravel and crushing equipment will continue to increase with the Beijing-Tianjin-Hebei integration, the Integrated Regional Development of Yangtze River Delta, the construction of Guangdong-Hong Kong-Macao Greater Bay Area and other large regional economic circles in China, the construction of urban residential buildings and large-scale national infrastructures in the future, and the construction of a large number of infrastructure projects under the Belt and Road Initiative [5]. Currently, there is a huge gap in river sand demand for infrastructure construction across China. In this context, importing river sand from neighboring countries is undoubtedly the best choice.

As an important river channel in the Philippines, the Santo Tomas River, which is home to the Pinatubo Volcano, is located in central Luzon and adjoins the South China Sea in the west. In 1991, the Pinatubo Volcano erupted and produced a large amount of lava. The lava covered the original riverbed and was mixed with the river sand, thus making the natural river sand there the riverbed’s unique geographical characteristics [6,7]. This paper studies and analyzes the properties of volcanogenic sand from the Santo Tomas River and prepares C30-C60 concrete made of it. In addition, this paper compares such C30-C60 concrete with concrete made of natural sand from Beijing in terms of workability, mechanical properties, and durability.

## 2. Materials and Methods

### 2.1. Materials

#### 2.1.1. Cementitious Materials

**Figure 1:**
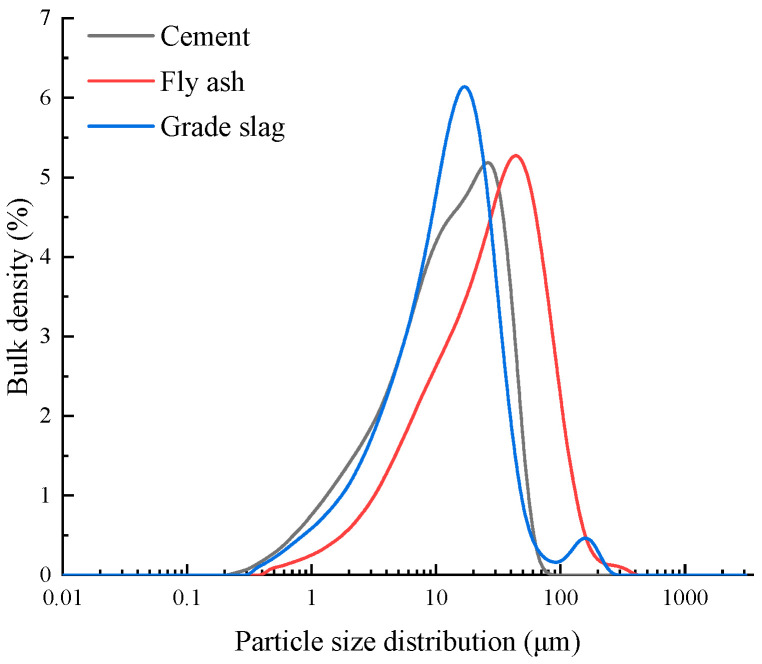
Particle size distribution of cementitious materials.

The cementitious materials used in this paper consist of cement, fly ash, and slag powder with a mass ratio of 3:1:1. P·O 42.5 ordinary Portland cement was used for the test in accordance with GB 175-2007 Common Portland Cement, which has a standard consistency water consumption of 27.8%, an 80 μm sieve residual percentage of 2.8%, a 28 d compressive strength, and a flexural strength of 52.2 MPa and 9.7 MPa, respectively. The chemical composition of cement is shown in Table 1. In accordance with GB/T 18046-2017 Ground granulated blast furnace slag used for cement, mortar, and concrete, grade-II fly ash has a 45 μm sieve residual percentage of 13.2%, a burning loss of 3.1%, and a water demand ratio of 102%. The chemical composition of grade-II fly ash is shown in Table 2. Furthermore, S95 grade slag has an activity index of 95% and a fluidity of 98%. The chemical composition of S95 grade slag is shown in Table 3. The particle size distribution of the cementitious material is shown in Figure 1.

#### 2.1.2. Aggregates

The performance indices of coarse aggregates prepared from crushed stones with particle sizes of 5–10 mm and 10–25 mm at a ratio of 1:4 are shown in Table 4. Natural sand from Beijing is used as the fine aggregate for the control group, which has a fineness modulus of 2.3 and belongs to Zone-II medium sand. The performance indices are shown in Table 5.

#### 2.1.3. Water-Reducing Agent and Water

The polycarboxylic acid water-reducing agent used for testing has a solid content of 10%, and a water reduction rate of 30%. In addition, the water used for the test was drinking water.

### 2.2. Mix Proportion of Concrete

The concrete mix proportion is shown in Table 6.

### 2.3. Test Methods

#### 2.3.1. Test Method of River Sand Performance

Refer to GB/T 14684-2022 Sand for construction, JGJ 52-92 Technical requirements and test methods of sand for ordinary concrete, and the JGJ 52-2006 Standard for technical requirements and test methods of sand and crushed stone (or gravel) for ordinary Concrete tests of the material properties of sand.

#### 2.3.2. Concrete Workability Test Method

Refer to GB/T 50080-2016 Standard for test method of performance on ordinary fresh concrete and GB/T 25181-2019 Ready-mixed Mortar to test the concrete mix performance.

#### 2.3.3. Test Methods for Mechanical Properties of Concrete

Refer to GB/T 50081-2016 Standard for test methods of mechanical properties on ordinary concrete, GB/T 50081-2019 Standard for test methods of concrete physical and mechanical properties, and GB/T 50107-2010 Standard for evaluation of concrete compressive strength to test the concrete’s strength. Non-standard specimens of 100 mm × 100 mm × 100 mm were cured under standard curing conditions until the ages of 3 d, 7 d, and 28 d, respectively. Their compressive strengths were determined in the unidirectional pressure loading mode. In addition, the obtained flexural strengths were multiplied by the dimensional conversion factor of 0.95. Non-standard specimens of 100 mm × 100 mm × 400 mm were cured under standard curing conditions until the age of 28 d. The flexural strength was determined by using a three-point bending loading method. The obtained flexural strength values were multiplied by the dimensional conversion factor of 0.85. The standard prismatic specimen of 150 mm × 150 mm × 300 mm was cured until the 28 d age under standard curing conditions, and the axial compressive strength was determined by unidirectional pressure action of loading.

#### 2.3.4. Concrete Durability Test Method

Refer to GB/T 50082-2009 Standard for test methods of long-term performance and durability of ordinary concrete, and JGJ/T 193-2009 Standard for inspection and assessment of concrete durability.

#### 2.3.5. Light-Matter Performance Test Method

Refer to GB/T 17431.1-2010 Lightweight Aggregates and its Test Method-Part 1: Lightweight Aggregates, GB/T 17431.2-2010 Lightweight Aggregates and its Test Method-Part 2: Test Methods for Lightweight Aggregates to test the light-matter performance.

## 3. Results

### 3.1. Material Properties of Volcanogenic Sand

**Figure 2:**
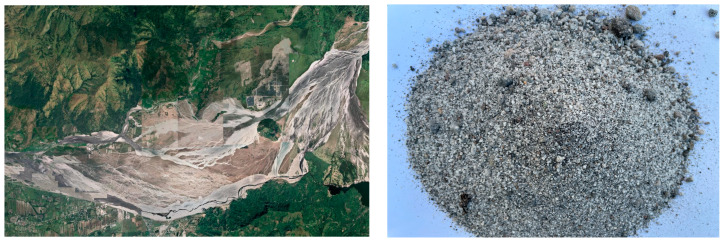
Geography of the Santo Tomas River, and volcanogenic sand.

**Figure 3:**
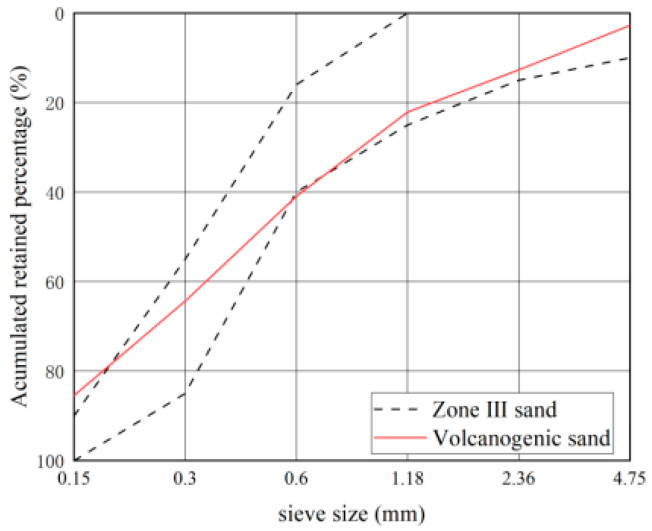
Gradation curve of volcanogenic sand.

The geographic location map of the Santo Tomas River and the volcanogenic sand in the Santo Tomas River area are shown in Figure 2, the gradation curves are shown in Figure 3, and the basic properties are shown in Table 7 and Table 8.

As seen in Figure 2 and Table 7 and Table 8, volcanogenic sand usually has a particle size of less than 0.6 mm and fails to meet the Zone-III requirements for gradation. As a result, it has a fineness modulus of only 2.2 and is deemed to be fine sand. In addition, its void ratio, mud content, and clay lump content are slightly higher than the values of no more than 44%, no more than 5%, and no more than 2% in GB/T 14684-2022, while its light-matter content is much higher than the values of no more than 2% specified in GB/T 14684-2022.

### 3.2. Workability of Concrete

**Figure 4:**
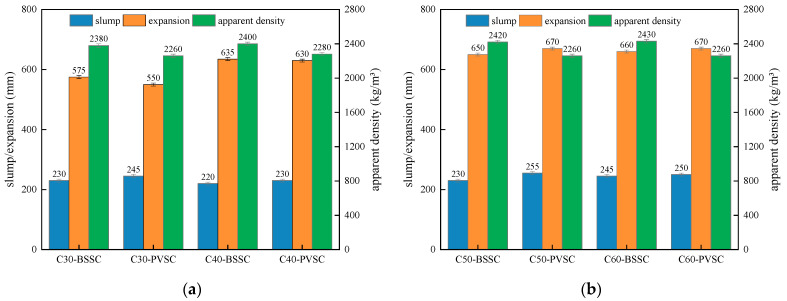
Workability of each concrete mix: (**a**) workability of C30/C40 concrete; (**b**) workability of C50/C60 concrete.

The workability of C30-C60 concrete with different sand sources is shown in Figure 4.

As seen in Figure 3, the slump of PVSC is basically the same as that of BSSC. However, compared to BSSC, PVSC has a poor slump-retaining capacity and a smaller apparent density. The poor slump-retaining capacity of PVSC is attributed to the fact that volcanogenic sand has a rough and porous surface with open-and-closed pores and a strong water absorption capacity [8]. Without pre-wetting, volcanogenic sand can absorb a large amount of water in a short period, thereby speeding up the slump loss of concrete within a short time and making it difficult to maintain the slump [9]. In addition, volcanogenic sand contains more light matter and has a smaller apparent density than volcanogenic sand. Additionally, due to the porous structure and small density of volcanogenic sand, PVSC has a smaller apparent density [10].

### 3.3. Mechanical Property Test of Concrete

#### 3.3.1. Test of Compressive Strength, Flexural Strength, and Axial Compressive Strength

**Figure 5:**
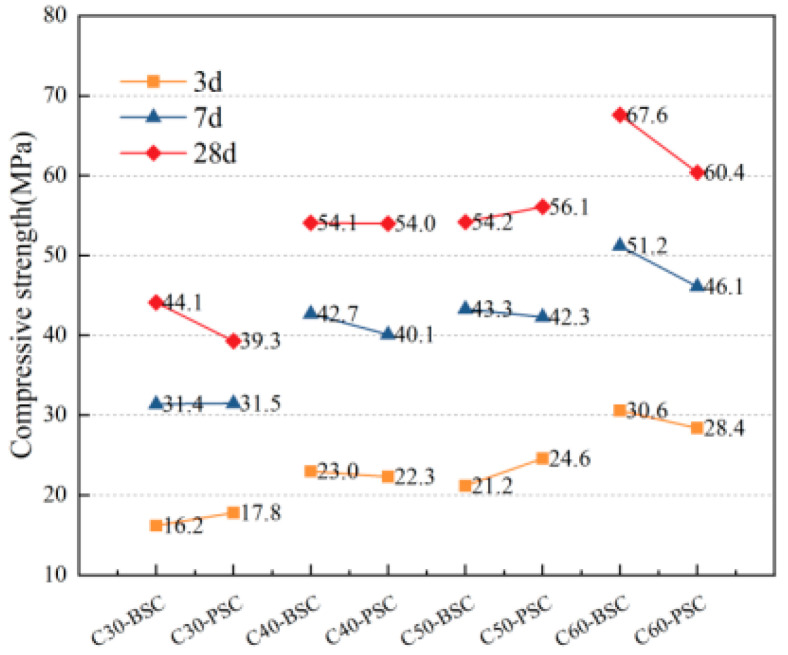
Compressive strengths of concrete with different ages and different sand sources.

**Figure 6:**
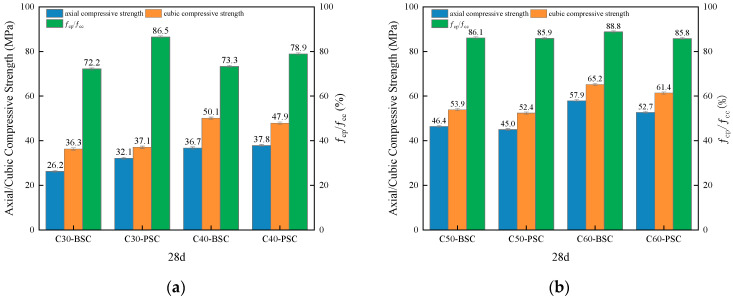
The 28 d axial compressive strengths of concrete with different sand sources: (**a**) Axial compressive strengths of C30 and C40 concrete; (**b**) Axial compressive strengths of C50 and C60 concrete.

**Figure 7:**
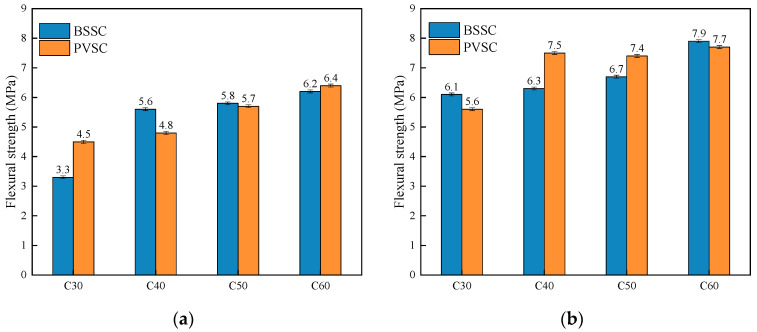
Flexural strength of concrete with different sand sources: (**a**) 28 d flexural strength; (**b**) 60 d flexural strength.

The compressive strengths of concrete of different strengths prepared with different fine aggregates at various ages are shown in Figure 5. The axial compressive strengths of concrete of different strengths prepared with different fine aggregates at various ages are shown in Figure 6. The flexural strengths of concrete of different strengths prepared with different fine aggregates at various ages are shown in Figure 7.

As shown in Figure 5, the compressive strengths of PVSC at different ages (3 d, 7 d, and 28 d) are basically the same as those of BSSC. Specifically, the 28 d compressive strength of C60-PVSC is slightly lower than that of BSSC. It can be seen from Figure 6 that the 28 d axial compressive strength of PVSC is basically the same as that of BSSC, and their *f*_cp_ and *f*_cc_ both range from 0.7 to 0.9, which approximate the generic values of *f*_cp_ and *f*_cc_ for light aggregate concrete [11]. In addition, as shown in Figure 7, PVSC and BSSC are not significantly different in the 28 d flexural strength and 60 d flexural strength, with a difference of only 0.1–1.2 MPa.

In fact, cement is an active component in concrete, and its strength directly affects the strength of the concrete made [12]. Results show that PVSC and BSSC have little difference in strength due to many reasons. First, the surface of Philippine volcanogenic sand is rough, loose, and porous. The sand can absorb water during the mixing process, thereby reducing the water-to-binder ratio and improving the compactness of cement stone. Second, the rough surface of volcanogenic sand particles improves the sand’s capacity for binding and bonding with cement stone, which makes the strength of light aggregate concrete improve [13].

#### 3.3.2. Elastic Modulus Test

The elastic modulus of concrete under static pressure is calculated according to the following formula:

(1) $$E_{C}=\frac{F_{a}-F_{0}}{A}\times \frac{L}{\Delta n}$$

(2) $$\Delta n=\varepsilon_{a}-\varepsilon_{0}$$

In the equation:

*E_c_*—Elastic modulus of concrete under static pressure (Unit: MPa).

*F_a_*—The stress shall be the load corresponding to one-third of the axial compressive strength (Unit: N).

*F*_0_—The stress shall be the initial load corresponding to 0.5 MPa (Unit: N).

*A*—The bearing area of the specimen (mm^2^).

*L*—Measuring range (mm).

∆*n*—Average value of deformation on both sides of the specimen when loading from *F*_0_ to *F_a_* for the last time (mm).

*ε_a_*—Average value of deformation on both sides of the specimen at *F_a_*.

*ε*_0_—Average value of deformation on both sides of the specimen at *F*_0_.

1 GPa = 1000 MPa.

**Figure 8:**
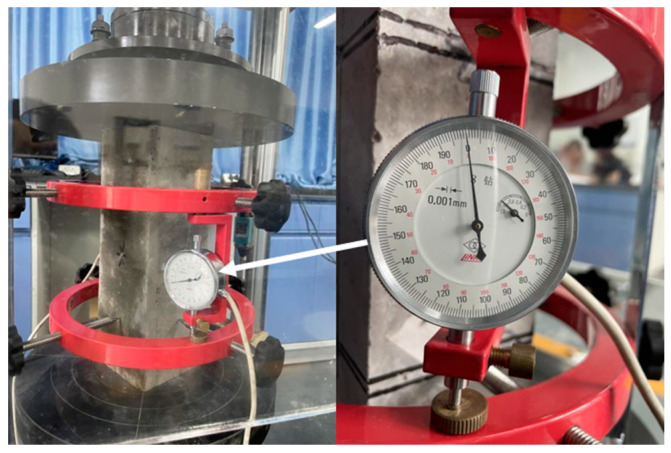
Elastic Modulus of Tester.

**Figure 9:**
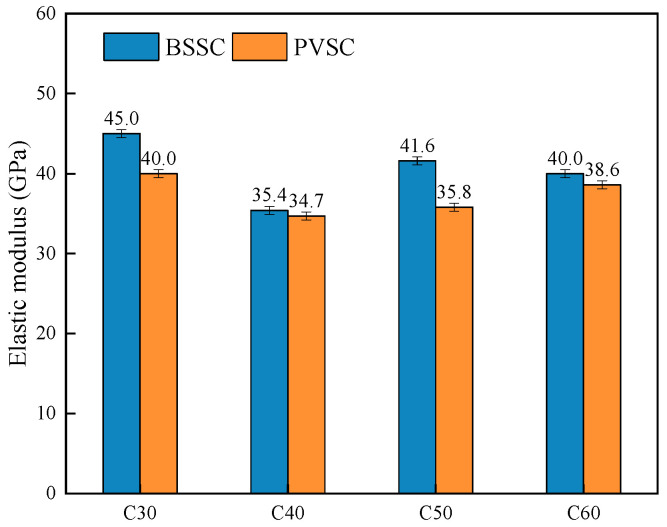
Elastic modulus of concrete with different sand sources.

The elasticity modulus tester is shown in Figure 8. The test results on the elastic modulus of concrete with different sand sources are shown in Figure 9.

As shown in Figure 9, the 28 d elastic modulus of C30-C60-PVSC is slightly lower than that of C30-C60-BSSC due to several reasons. First, in terms of fineness modulus and gradation, Philippine volcanogenic sand is inferior to the natural river sand in Beijing. Second, volcanogenic sand has a porous structure that absorbs some of the water and reduces the water-binder ratio around the aggregate [14].

#### 3.3.3. Drying Shrinkage Test

**Figure 10:**
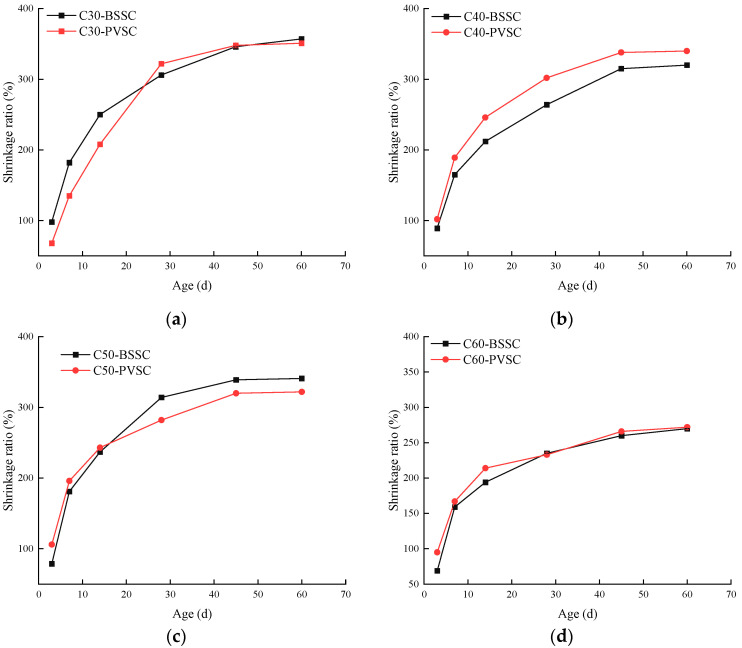
Drying shrinkage of concrete with different sand sources: (**a**) drying shrinkage of C30 concrete; (**b**) drying shrinkage of C40 concrete; (**c**) drying shrinkage of C50 concrete; (**d**) drying shrinkage of C60 concrete.

A horizontal concrete shrinkage meter was used to determine the dry shrinkage deformation at different ages, with pre-embedded copper probes at both ends of the specimens. The experimental results are shown in Figure 10.

As shown in Figure 10, the drying shrinkage of concrete decreases with the increase of concrete strength, because the amount of fine aggregate decreases with the increase of concrete strength [15], i.e., the content of stone dust in concrete also decreases. At an earlier age, the drying shrinkage rate of PVSC is greater than that of BSSC. However, as the age is extended, the drying shrinkage rate of PVSC increases gently, and eventually approximates that of BSSC. Due to a higher content of stone dust in volcanogenic sand than that of natural river sand in Beijing, the slurry content in PVSC increases in the early stages, thereby resulting in an increase in the shrinkage thereof. At a later stage, the filling effect of stone dust improves the concrete’s compactness and inhibits its shrinkage. Meanwhile, the water required for concrete hardening is guaranteed with an increase in water demand, thereby reducing the shrinkage of concrete and causing the effect of internal curing [16,17].

### 3.4. Concrete Durability Test

#### 3.4.1. Test of Chloride Ion Penetration Resistance of Concrete

In this paper, the NEL method was used to test the chloride ion penetration resistance of concrete. The NEL method assumes that the particle concentration is the chloride ion concentration of concrete hole solution or the used salt solution concentration. On the basis of measuring the conductivity of concrete after salt-filling, the diffusion coefficient of chloride ions in concrete is calculated by using the Nernst–Einstein equation [18]. This method enables us to determine the chloride ion diffusion coefficient of C20-C100 concrete within 5–8 min, thereby truly realizing the rapid evaluation of concrete durability. This method conforms to the ASTM C1202 United States standard.

**Figure 11:**
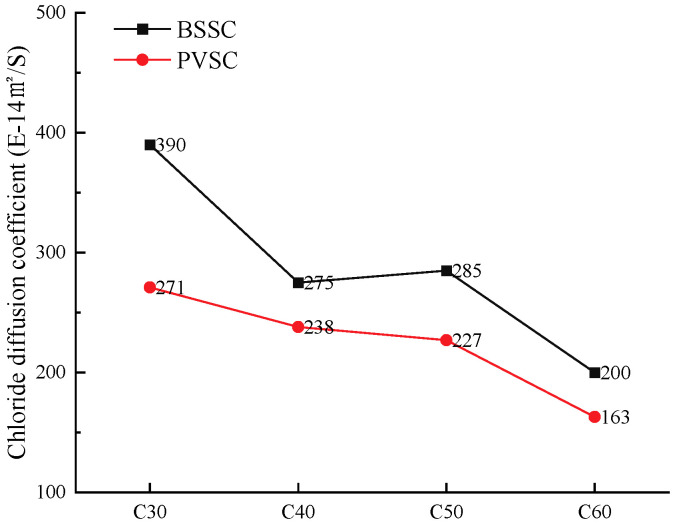
Diffusion coefficients of chloride ions in concrete with different sand sources and relevant evaluation.

The evaluation criteria of the NEL method are shown in Table 9, and the test results are shown in Figure 11.

As shown in Figure 11, the chloride ion diffusion coefficient of PVSC is lower than that of BSSC with the same strength, while PVSC has a better resistance to chloride ion penetration. Concrete formulated with Philippine volcanogenic sand had 30.5%, 13.4%, 20.3%, and 18.5% lower chloride ion diffusion coefficients compared to concrete formulated with the same grade of Beijing natural river sand, respectively. The surfaces of the lightweight particles in volcanogenic sand are rough and porous, so the hydration products of cementitious materials can penetrate into the lightweight particles of volcanogenic sand. Due to the internal curing effect, the water inside the lightweight particles of river sand is released and participates in the hydration reaction of cement, which increases the compactness of the concrete structure and helps to prevent the transference of chloride ions [19].

#### 3.4.2. Concrete Carbonation Resistance Test

**Figure 12:**
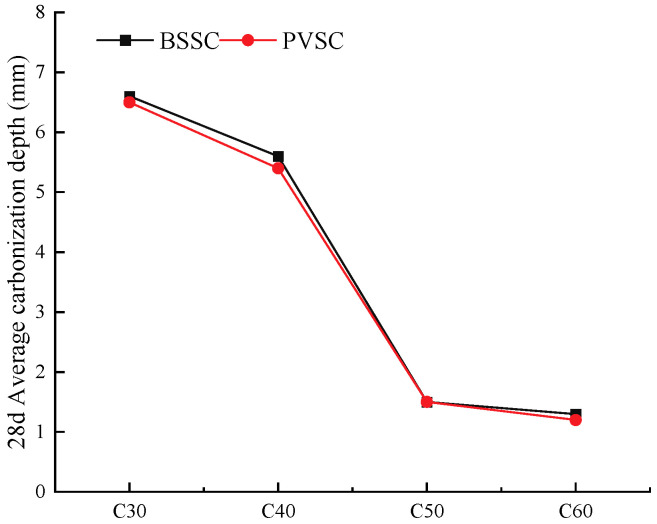
Carbonation of concrete of different strengths.

During the concrete carbonation test, the cubic specimens were cured for 26 d under standard curing conditions, then dried in a vacuum oven at 60 °C for 2 d, and placed under a rapid carbonation chamber for rapid carbonation immediately after cooling down. The concentration of carbon dioxide in the box is maintained at (20 ± 3) %, the relative humidity is controlled at (70 ± 5) %, and the temperature is controlled at (20 ± 2) °C. Notably, 28 d later, they were split to determine the carbonation depth using phenolphthalein. The results of the concrete carbonation test are shown in Figure 12.

As shown in Figure 12, the 28 d carbonation depths of C30-C60-PVSC range from 1.2 mm to 6.6 mm, which are basically the same as those of C30-C60-BSSC. When PVSC is exposed to air, CO_2_ gradually diffuses from its surface to the interior of the concrete through the capillary pore channels between cement stones, pores in cement stones and aggregates, as well as microcracks and small pores. In addition, the porous surface of volcanogenic sand also promotes CO_2_ infiltration. However, water absorption by volcanogenic sand in porous structures reduces the water-to-binder ratio and promotes the formation of a compact “self-vacuum” protective layer at the transition zone of interface with cement stone [20]. As a result, the rapid infiltration of CO_2_ is hindered, and the early carbonation resistance of PVSC is enhanced. It is also possible that the concrete structure becomes more compact due to the filling effect of stone dust in the volcanogenic sand and the improvement of the interfacial transition zone between the slurry and the aggregates [21].

#### 3.4.3. Concrete Seepage Resistance Test

**Figure 13:**
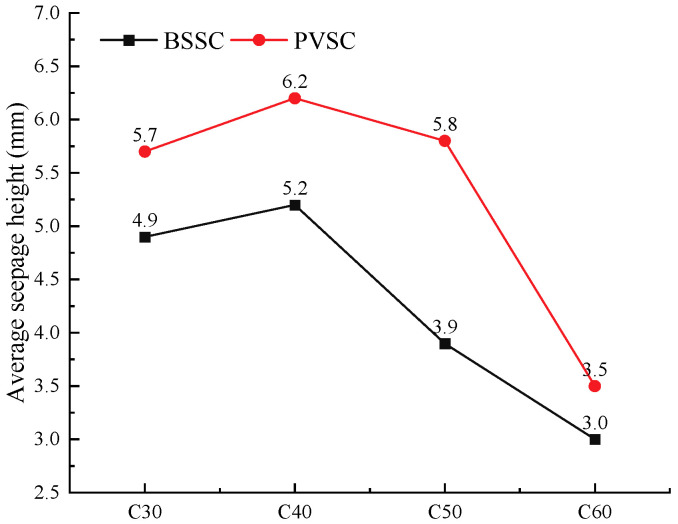
Concrete penetration depth.

For the purpose of the test, the average seepage height was determined based on the constant water pressure, which is used to express the concrete seepage resistance. Test results are shown in Figure 13.

As shown in Figure 13, PVSC is inferior to BSSC in terms of seepage resistance due to several reasons. First, volcanogenic sand has many pores, particularly open pores, which cause an increase in pathways for water infiltration. Second, the surface area of volcanogenic sand is large, and more cement mortar is needed to wrap the aggregates. Therefore, a lack of cement mortar will result in a poor effect of concrete wrapping, a decrease in the bonding area of the interface between the aggregate and the slurry, and the formation of water seepage pores in the concrete interface area, further resulting in poor seepage resistance [22,23].

### 3.5. Optimization

#### 3.5.1. Material Properties of Optimized Volcanogenic Sand

**Figure 14:**
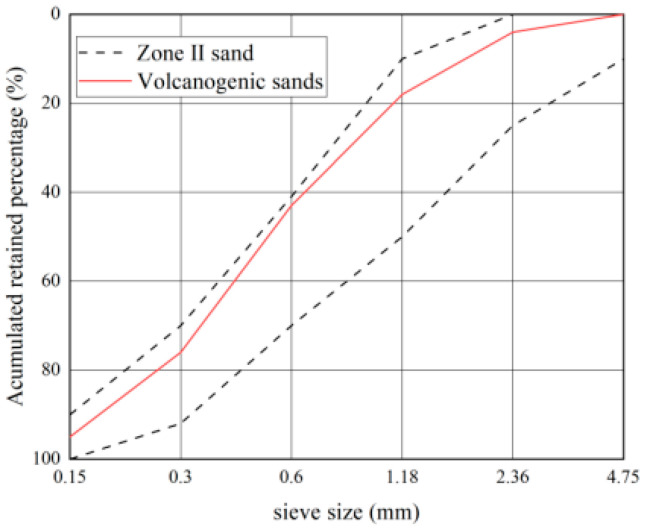
Gradation curves of optimized volcanogenic sand.

Table 10 and Table 11 and Figure 14 show the optimized volcanogenic sand.

As shown in Table 10 and Table 11 and Figure 14, the optimized volcanogenic sand has a fineness modulus of 2.4 and a specific particle size of 7.1. According to the Chinese standard GB/T 14684-2022, the volcanogenic sand is classified as medium sand. The apparent density and bulk density of the optimized volcanogenic sand increased to 2570 kg/m^3^ and 1550 kg/m^3^, respectively, while the void ratio decreased to 40%. After optimization, substandard volcanogenic sand became qualified in all three parameters. However, it is still unqualified in light-matter content and belongs to natural volcanogenic light sand, which is different from that specified in GB/T 14684-2022. Without adverse effects on various properties of concrete, the optimized volcanogenic sand can meet the requirements for ordinary engineering.

#### 3.5.2. Workability and Compressive Strength of Optimized PVSC

Due to a high proportion of lightweight large particles, the volcanogenic sand is unqualified in many test indices. In view of this, such lightweight large particles therein were separated out and washed according to Chinese standard GB/T 14684-2022 to obtain the optimized volcanogenic sand and massive light matter containing large particles. Whereas the composition of volcanogenic sand remains unchanged before and after optimization, This paper tests the material properties of the optimized volcanogenic sand, the workability and compressive strength of the new concrete for the unqualified items in volcanogenic sand, so as to eliminate the non-conformity of volcanogenic sand.

The proportions of the mix of concrete made from the optimized volcanogenic sand are shown in Table 12.

**Figure 15:**
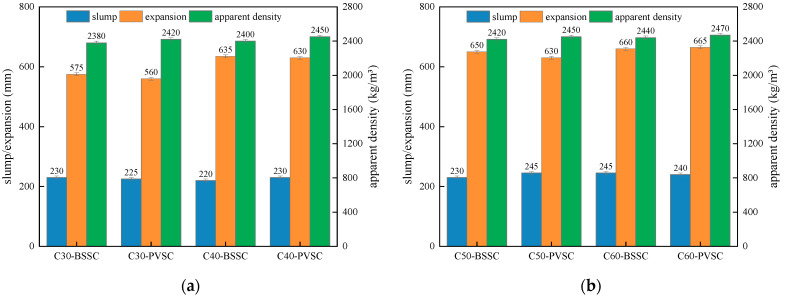
Workability of each group of concrete after optimization: (**a**) Workability of C30 and C40 concrete; (**b**) Workability of C50 and C60 concrete.

**Figure 16:**
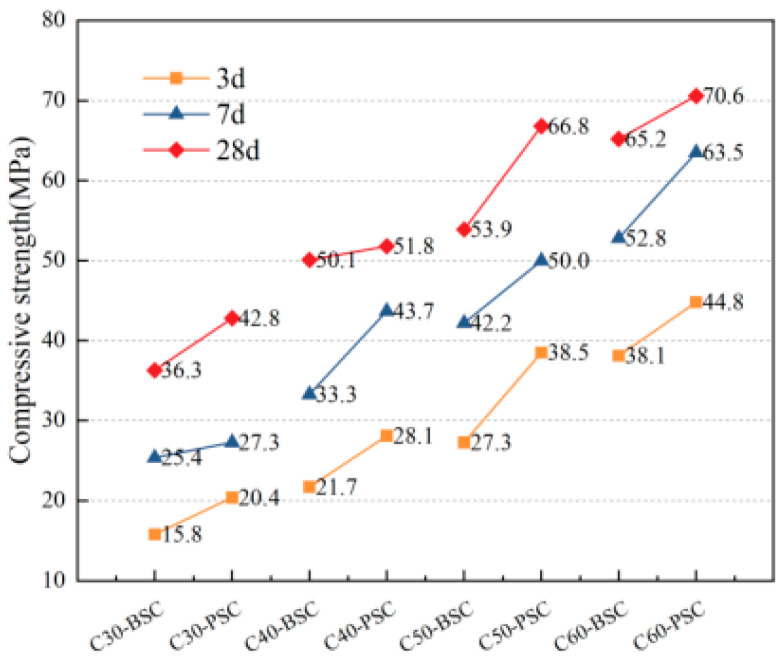
Compressive strengths of PVSC and BSSC after optimization.

The workability of C30-C60 concrete with different sand sources is shown in Figure 15. The compressive strengths of C30-C60 concrete with different sand sources at various ages are shown in Figure 16.

As shown in Figure 15 and Figure 16, PVSC is superior to BSSC in terms of compressive strength after optimization, while they are basically the same in terms of slump, expansion, and apparent density. There exist massive open pores and closed pores inside the lightweight large particles of volcanogenic sand, which results in lighter mass, lower strength, and stronger water absorption [24]. After the sieving of such particles, the water absorption of the volcanogenic sand was inhibited, which improved the poor slump-retaining capacity of fresh concrete as well as the apparent density and compressive strength of PVSC.

### 3.6. Light-Matter Research

**Figure 17:**
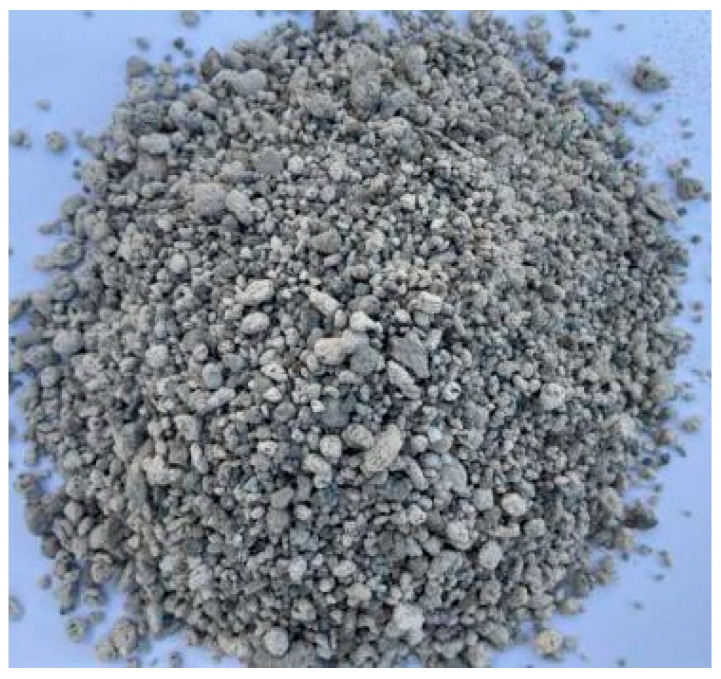
Light-matter particles.

The chemical composition and material properties of the separated light-matter particles are shown in Figure 17.

#### 3.6.1. Chemical Composition of Light-Matter

X-ray fluorescence (XRF) tests were conducted after drying and grinding separated light matter. The test results are shown in Table 13.

As shown in Table 13, the light matter separated from the Philippine volcanogenic sand is basically the same as the Philippine volcanogenic sand in the chemical composition. It indicates that they are the gravel of larger particle sizes in the volcanogenic sand.

#### 3.6.2. Light-Matter Properties

**Figure 18:**
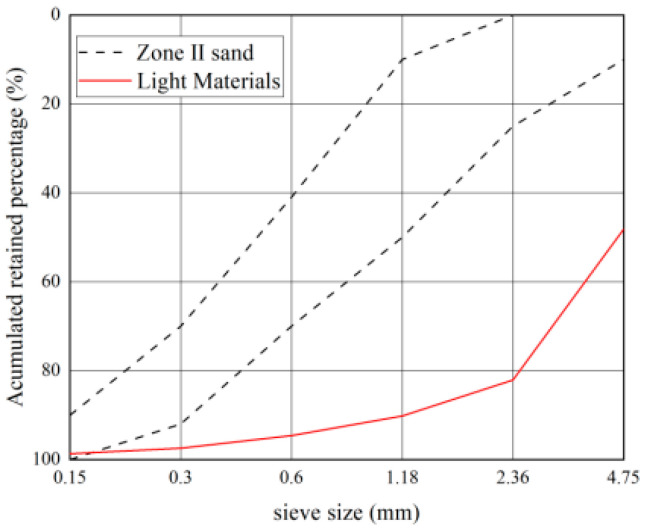
Gradation curves of light matter in volcanogenic sand.

The particle gradation of lightweight large particles and fine aggregates after sieving for the test is shown in Table 14 and Table 15, and the gradation curve is shown in Figure 18.

The optimized light matter as natural light aggregates of volcanic cinder has a bulk density of 960 kg/m^3^, which is less than 1200 kg/m^3^. According to the gradation curves of light matter, the light matter in Philippine volcanogenic sand does not meet the gradation requirements in GBT 14684-2022. In addition, the light matter in Philippine volcanogenic sand fails to meet China’s gradation requirements for light aggregates.

#### 3.6.3. Performance Test of Lightweight Mortar

Some mortar with the strength grade M30 made of natural sand from Beijing and volcanogenic sand, respectively, was prepared. Their mix proportion is shown in Table 16, and the test results are shown in Table 17.

As shown in Table 16 and Table 17, under the condition of the same cementitious material and water consumption, the water-reducing agent dosage of mortar prepared with light matter from Philippine volcanogenic sand is lower than that of mortar prepared with Beijing natural sand. Specifically, only 0.8% of the water-reducing agent is needed to achieve a consistency of 84 mm. In addition, comparison results show that the apparent density of the mortar prepared with the light matter in the volcanogenic sand is only 84% of that of mortar prepared with natural sand from Beijing. However, the water-retention rate of the former is 92%, which is higher than that of the latter [25,26]. The 7 d compressive strength of volcanogenic sand light aggregate mortar can reach 30.2 MPa.

## 4. Discussion

The detection age for concrete durability performance indicators specified in the Chinese national standard GB/T 50082-2009 is usually tens of days, while the service life of concrete in practical engineering is a dozen years or even decades. Although the performance of volcanic sand concrete is not much different from that of ordinary concrete in a short term, the long-term durability needs to be further observed. Specific test results show that volcanogenic sand can be used to prepare C30-C60 grade concrete, but it is still uncertain whether it can be used to prepare high-strength concrete or ultra-high-strength concrete. At the same time, although the light substance content is greatly reduced after optimization, it is still higher than the value of not more than 2% specified in the national standard GB/T 14684-2022. If the light substance content can be further reduced to meet the requirement, the utilization rate of volcanogenic sand will be further improved.

## 5. Conclusions

This paper analyzes the material properties of Philippine volcanogenic sand, as well as the workability, mechanical properties, and durability of PVSC. The conclusions are drawn as follows:

- PVSC is good in workability and resistance to chloride ion penetration and carbonation but is weak in seepage resistance. Additionally, it has a slightly low apparent density and a strength of up to C30-C50. Compared to BSSC, PVSC is similar in overall performance but has a large slump loss.
- Philippine volcanogenic sand contains as much as 12.2% of light matter. The volcanogenic sand optimized through sieving, washing, and drying large particles of more than 4.75 mm reaches the standard of Zone-II medium sand, which obtains a fineness modulus of 2.4, an apparent density of 2570 kg/m^3^, a bulk density of 1550 kg/m^3^, and a light-matter content of 6.0%. Compared with BSSC of the same grade, PVSC, after optimization 1., is basically the same in terms of slump, expansion, and apparent density and has a good slump-retaining capacity and 2. higher compressive strength.
- The separated light matter has a fineness modulus of 4.3, a cylindrical compressive strength of 2.8 MPa, a 1 h water absorption rate of 8.2%, and a bulk density of 960 kg/m^3^. Under the condition of the same cementitious material and water consumption, the mortar made of Philippine volcanogenic sand, compared to the mortar prepared with natural sand from Beijing, has a low apparent density of 1940 kg/m^3^ and a water-retention rate of 92%, and the seven-day compressive strength is 30.2 MPa.

## Figures and Tables

**Table 1:** The main chemical composition of cement (%).

Name	CaO	SiO_2_	Al_2_O_3_	Fe_2_O_3_	MgO
Cement	64.60	21.30	6.14	4.42	1.94

**Table 2:** The main chemical composition of fly ash (%).

Name	SiO_2_	Al_2_O_3_	Fe_2_O_3_	CaO	K_2_O	SO_3_	MgO	Na_2_O
Grade-II fly ash	50.23	33.22	5.95	4.55	1.48	0.91	0.79	0.47

**Table 3:** The main chemical composition of grade slag (%).

Name	CaO	SiO_2_	Al_2_O_3_	MgO	SO_3_	Fe_2_O_3_	K_2_O	Na_2_O
S95 grade slag	38.44	31.86	16.22	6.96	1.98	1.79	0.62	0.56

**Table 4:** Performance indexes of coarse aggregates of two grades (%).

Grain Composition (mm)	Bulk Density (kg/m^3^)	Mud Content	Clay Lump	Crush Index	Void Ratio
10–20	2870	0.3	0.0	5.4	45
5–10	2840	0.2	0.0	/	44

**Table 5:** Basic properties of natural sand from Beijing.

Fineness Modulus	Specific Granularity	Apparent Density (kg/m^3^)	Bulk Density	Void Ratio (%)
2.3	7.2	2630	1820	31

**Table 6:** Mix proportion of Beijing sand source concrete (BSSC) and Philippine volcanic sand concrete (PVSC) kg/m^3^.

Sample	Cement	Fly Ash	Slag Powder	Water	Fine Aggregate	Coarse Aggregate	Water Reducer	Sand Ratio (%)
C30-BSSC	218	73	73	180	804	990	1.74%	44
C40-BSSC	260	87	87	182	682	1023	1.80%	40
C50-BSSC	300	100	100	175	642	1047	1.80%	38
C60-BSSC	317	106	106	159	613	1090	1.80%	36
C30-PVSC	218	73	73	180	698	980	1.60%	42
C40-PVSC	260	87	87	182	677	1015	1.80%	42
C50-PVSC	300	100	100	175	615	1014	1.80%	38
C60-PVSC	317	106	106	159	591	1051	2.05%	36

**Table 7:** Basic properties of volcanogenic sand (%).

Fineness Modulus	Specific Granularity	Apparent Density (kg/m^3^)	Bulk Density (kg/m^3^)	Void Ratio	Mud Content	Clay Lump
2.2	6.3	2380	1320	45	6.7	2.2

**Table 8:** Other attributes of volcanogenic sand.

Chlorine Ion Content	Organic Content	Soundness	Light Matter	Methylene Blue
0.00071%	qualified	Ⅰ	12.2%	1.2

**Table 9:** Evaluation criteria of NEL method.

Chloride Diffusion Coefficient (10–14 m^2^/s)	Permeability
>1000	Ⅰ (taunt)
500–1000	Ⅱ (high)
100–500	Ⅲ (middle)
50–100	Ⅳ (low)
10–50	Ⅴ (very low)
5–10	Ⅵ (ultra Low)
<5	Ⅶ (ignore)

**Table 10:** Basic properties of optimized volcanogenic sand (%).

Fineness Modulus	Specific Granularity	Apparent Density	Bulk Density	Void Ratio	Mud Content
2.4	7.1	2570 kg/m^3^	1550 kg/m^3^	40	1.7

**Table 11:** Other properties of volcanogenic sand after optimized treatment.

Organic Content	Soundness	Light Matter	Methylene Blue
qualified	Ⅰ	6.0%	1.0

**Table 12:** Mix proportions of optimized PVSC kg/m^3^.

Sample	Cement	Fly Ash	Slag Powder	Water	Sand	Coarse Aggregate	Water Reducer	Sand Ratio (%)
C30-PVSC	218	73	73	180	698	980	1.31%	42
C40-PVSC	260	87	87	182	677	1015	1.44%	40
C50-PVSC	300	100	100	175	615	1014	1.67%	38
C60-PVSC	317	106	106	159	591	1051	2.05%	36

**Table 13:** Chemical composition of different samples (%).

Chemical Composition	SiO_2_	Al_2_O_3_	CaO	Na_2_O	Fe_2_O_3_	K_2_O	MgO	Loss on Ignition
Volcanogenic sand	62.66	18.23	6.61	4.74	3.87	1.43	1.38	0.7
Light-matter particle	67.39	14.85	5.15	3.23	4.60	2.53	1.17	0.8

**Table 14:** Gradation of light-matter particles.

Mesh Size (mm)	4.75	2.36	1.18	0.6	0.3	0.15	Mesh Ground
**Grader retained percentage (%)**	48.17	33.91	8.09	4.43	2.83	1.24	0.99

**Table 15:** Basic properties of light matter.

Fineness Modulus	Specific Granularity	Cylindrical Compressive Strength	1 h Water Absorption	Bulk Density	Density Grade
4.3	1.1	2.5 MPa	8.2%	960 kg/m^3^	1000

**Table 16:** Mix proportion of Beijing sand source mortar (BSSM) and Philippine volcanic sand mortar (PVSM) kg/m^3^.

Sample	Cement	Sand	Water	Water Reducer
BSSM	480	1819	270	1.1%
PVSM	480	1086	270	0.8%

**Table 17:** Mortar test results.

Sample	Consistency (mm)	Water-Retention rate	Apparent Density (kg/m^3^)	7 d Strength (MPa)
BSSM	70	89%	2300	31.5
PVSM	84	92%	1940	30.2

## Data Availability

Not applicable.

## Abbreviations

Beijing Sand Source Concrete (BSSC); Philippine Volcanogenic Sand Concrete (PVSC). Beijing sand source mortar (BSSM); Philippine volcanic sand mortar (PVSM).
